# Characterization of Behavioral Phenotypes in Heterozygous DAT Rat Based on Pedigree

**DOI:** 10.3390/biomedicines11092565

**Published:** 2023-09-18

**Authors:** Gioia Zanfino, Concetto Puzzo, Vincenzo de Laurenzi, Walter Adriani

**Affiliations:** 1 Center for Behavioral Sciences and Mental Health, Istituto Superiore di Sanità, 00161 Rome, Italy; zanfinogioia@gmail.com (G.Z.); concetto.puzzo@guest.iss.it (C.P.); 2 Faculty of Psychology, International Telematic University Uninettuno, 00186 Rome, Italy; 3 Center for Advanced Studies and Technology (CAST), “G. d’Annunzio” University of Chieti-Pescara, 66100 Chieti, Italy; vincenzo.delaurenzi@unich.it; 4 Department of Innovative Technologies in Medicine and Dentistry, “G. d’Annunzio” University of Chieti-Pescara, 66100 Chieti, Italy

**Keywords:** ADHD, OCD, transgenerational inheritance, epigenetic effects

## Abstract

Dopamine is an essential neurotransmitter whose key roles include movement control, pleasure and reward, attentional and cognitive skills, and regulation of the sleep/wake cycle. Reuptake is carried out by the dopamine transporter (DAT; *DAT1 SLC6A3* gene). In order to study the effects of hyper-dopaminergia syndrome, the gene was silenced in rats. DAT-KO rats show stereotypical behavior, hyperactivity, a deficit in working memory, and an altered circadian cycle. In addition to KO rats, heterozygous (DAT-HET) rats show relative hypofunction of DAT; exact phenotypic effects are still unknown and may depend on whether the sire or the dam was KO. Our goal was to elucidate the potential importance of the parental origin of the healthy or silenced allele and its impact across generations, along with the potential variations in maternal care. We thus generated specular lines to study the effects of (grand) parental roles in inheriting the wild or mutated allele. MAT-HETs are the progeny of a KO sire and a WT dam; by breeding MAT-HET males and KO females, we obtained subjects with a DAT -/- epigenotype, named QULL, to reflect additional epigenetic DAT modulation when embryos develop within a hyper-dopaminergic KO uterus. We aimed to verify if any behavioral anomaly was introduced by a QULL (instead of KO) rat acting as a direct father or indirect maternal grandfather (or both). We thus followed epigenotypes obtained after three generations and observed actual effects on impaired maternal care of the offspring (based on pedigree). In particular, offspring of MAT-HET-dam × QULL-sire breeding showed a compulsive and obsessive phenotype. Although the experimental groups were all heterozygous, the impact of having a sire of epigenotype QULL (who developed in the uterus of a KO grand-dam) has emerged clearly. Along the generations, the effects of the DAT epigenotype on the obsessive/compulsive phenotype do vary as a function of the uterine impact on either allele in one’s genealogical line.

## 1. Introduction

Dopamine is a catecholaminergic neurotransmitter involved in numerous functions in both the central and peripheral nervous systems. Dopamine plays essential roles in the regulation of the sleep–wake cycle, motor control, working memory [1], the reward system [2], and motivation [3]. The search for novelty, food eating, engaging in physical activity, or similar rewarding activities, as well as taking drugs of abuse, increase dopamine levels. This increase occurs not only when the activity being performed is more rewarding than expected but also when predicting or anticipating future rewards [4]. Dopamine is also involved in maternal behavior, especially mediating individual differences in maternal care [5,6,7]. In fact, previous studies in rats demonstrated secreted dopamine in the nucleus accumbens (NAcc) of mothers in response to their pups [7,8,9,10,11,12,13]. Maternal care is important for optimal offspring development and any even-slight impairment has major long-term outcomes [14].

Once released, dopamine affects a large area [15,16]. For this reason, systems designed to remove the neurotransmitter regulate dopamine’s extracellular concentrations. Among these systems, the dopaminergic transporter (DAT), encoded by the *SLC6A3* gene, plays a significant role. Alterations in DAT are linked with a variety of neuropsychiatric disorders, such as attention deficit/hyperactivity disorder (ADHD), obsessive-compulsive disorder (OCD) [17], schizophrenia [18], autism spectrum disorder (ASD) [19,20,21], and also drug addiction [22].

DAT-KO rats exhibit hyperactivity, impulse control, and stereotypes; KO females are unable to produce milk and to take care of their offspring [23,24]. Here, we aim to expand our knowledge of the heterozygous (DAT-HET) model. The only functioning allele available could be sensitive to potential epigenetic modulations [25]. In fact, the phenotype shown by heterozygous rats varies based on the maternal or paternal origin of the healthy or mutated allele [26,27]. Many recent studies have demonstrated that stressful conditions that are experienced by parents can influence the offspring’s vulnerability to many pathological conditions, including psychopathologies—primarily related to a disruption in stress response mechanisms. These effects may even endure for several generations [28]. Therefore, as dopaminergic dysregulation is a key element in psychiatric disorders, our objective is to investigate the effects of transmitting potential epigenetic modulations present on DAT (the dopamine transporter) across various generations. These modulations, in combination with environmental factors, can lead to susceptibility to developing psychological disorders.

From a translational standpoint, our model aims not only to characterize the importance of intergenerational transmission but also the indirect impact of DAT-related behavioral changes via maternal care. This intergenerational transmission may be caused by the interplay of social and genetic factors [29], intertwining closely the two components: on one side, the putative epigenetic marks on the DAT allele may alter the dynamics of the dopaminergic system, hence altering maternal behavior; on the other hand, such altered cares during infancy will shape the phenotype of females, which will then become dams, thereby impacting on second-generation offspring. For this reason, the possibility to study up to four different generations in the laboratory allows us to characterize the distinct behavioral phenotypes in relation to the localization, in the pedigree, of the epigenetic mark represented in our hands by intrauterine development under hyperdopaminergia. In fact, childhood trauma is a precursor to a variety of psychological disorders such as anxiety, mood instability, and personality changes [30].

Childhood maltreatment also increases the likelihood of developing evident mental disorders in adulthood [31], with a particularly high incidence of obsessive-compulsive disorder [30,32]. It can be hypothesized that parents who mistreat their children may transfer this behavioral pattern to them, as they are prone to replicating what they themselves endured as adults. Children who have been maltreated by their parents are more likely to perpetuate the same behavior in the next generation [33,34].

### 1.1. State of the Art

In a recent study [35], MAT-HET subjects where the letter M indicates the healthy allele from the mother (offspring of a WT female and a KO male) were compared to PAT-HET subjects where the letter P indicates the healthy allele from the father (offspring of a KO female and a WT male). In the first generation, the mutated allele from the father to MAT-HET matures in the hyperdopaminergic epididymis before the zygote is conceived; vice versa, in PAT-HET, the egg (as well as the embryo and fetus) matures in the hyperdopaminergic uterus. These environmental factors may explain the epigenotypes as being due to a putative allele’s epigenetic imprint. In addition, a second and a third generation have been generated to determine if the transgenerational inheritance affecting behavior could be related to the specular initial ancestor. Significant transgenerational epigenetic effects have been observed: intense locomotor activity was observed in the generation from MAT-HET dam × PAT-HET sire, and decreased locomotor activity was found in the generation from PAT-HET dam x MAT-HET sire. At the third generation, the circadian cycles normalize, reaching values similar to those of WT [35].

To investigate the transgenerational epigenetic impact on behavioral phenotype, we crossed DAT rats in various combinations and evaluated the effects associated with location in the pedigree of the original DAT -/- allele (also exploiting taxonomy codes to keep track across the generations). From the breeding between MAT-HET and KO parents, we obtained specular heterozygotes depending on who was the sire and who was the dam. When a KO sire mated with a MAT-HET dam, we obtained offspring where half had the KO genotype and half had the MIX-HET genotype [36]. When conversely, we bred a MAT-HET male with a KO female [26,37], we obtained half of the offspring with a heterozygous epigenotype (named MUX-HET, a sort of MIX-HET but instead born from a KO dam, namely born from hyperdopaminergic uterus) and the other half with a somewhat innovative DAT -/- epigenotype (a sort of KO but additionally also born from hyperdopaminergic uterus, therefore named QULL). In both offspring, the letter U indicates development in a hyperdopaminergic uterus [36]. A QULL epigenotype deserves particular attention because it is genetically devoid of DAT but also developed in a hyperdopaminergic uterus within a KO dam. This excess of dopamine, both the own-released one and that coming from the placenta, can further aggravate the offspring’s epigenotype, notably while gonads are developing in the embryo. Therefore, we obtained different types of heterozygous rats, each with their own characteristic marks from a QULL ancestor, which allowed us to evaluate the epigenetic effects of inheriting the healthy or mutated allele from specular branches of the pedigree.

### 1.2. Aims of This Study

Our aim in exp. 1 was to assess the maternal behavior of MAT-HET and µAT-HET epigenotype dams, assuming that, although they both are in heterozygous condition, they may exhibit different behavioral phenotypes: while MAT-HET is a reference group (from WT dam x KO sire), µAT-HET dams are the offspring of a QULL father and a WT mother. Now, data suggest the presence of impairments in maternal care from µAT-HET due to epigenetic modulations inherited from a QULL instead of KO sire and an anomalous behavioral phenotype in the offspring.

In the second generation, we observed behavioral phenotypes in three groups consisting of MIX-HET (offspring of a MAT-HET female and a KO male), a second group consisting of µIX-HET (offspring of a µAT-HET female and a KO male), and a third group consisting of NIX-HET (offspring of a MAT-HET mother and a QULL father). In all epigenotypes, the healthy allele comes from the maternal lineage, whereas the mutated allele comes from the paternal lineage. On top of all this, our design allowed us to compare the putative epigenetic marks of a QULL gamete coming from the direct father (in NIX-HET) or the maternal grandfather (in µIX-HET); for that reason, we decided to further investigate the combination of both transgenerational inheritances by adding a vIX group (offspring of a µAT-HET female and a QULL male).

In conclusion, we evaluated the behavioral phenotype observed in DAT-HET in relation to the transmission of the healthy or mutated allele, which can carry epigenetic modulations developed in a hyperdopaminergic uterus. We further assume that these modulations may differ if the inherited allele comes from an egg or a spermatozoon.

## 2. Methods

To study the role of DAT in various diseases, transgenic animal models with deficient DAT function have been created by Leo and her group (2018). In this model, using biotechnologies, a stop codon was inserted into the structure of the *SLC6A3* gene encoding for the DAT protein. In this way, DAT is still expressed but truncated to less than 70 amino acids, resulting in an ineffective protein. In DAT-KO rats, the *SLC6A3* gene is produced but functionally silenced. DAT-KO rats exhibit hyperactivity, issues of impulse control, and stereotypes. Additionally, both male and female KO are fertile, but KO females show an inability to produce milk, impaired maternal behavior, and inadequacy to take care of their offspring [23,24].

All rats were kept, according to an inverted light–dark cycle (lights off at 8 a.m. and on at 8 p.m.), in a room with controlled temperature and humidity (T 21 ± 1 °C; relative humidity 60 ± 10%) and with food and water ad libitum. The series of experiments started with mating and breeding, in which maternal behavior was evaluated, and then the offspring were tested shortly after weaning, to study the effects of the different epigenotypes.

The experimental subjects were DAT-HET female rats and their postnatal pups. Same-epigenotype females were housed in pairs, and a male was introduced, which was later removed when both females, after about two weeks, showed weight gain and abdominal enlargement. We used a male for every two females, i.e., three KO males for six μAT-HET females (offspring μIX); three KO males for six MAT-HET females (offspring MIX-HET); and three QULL males for six MAT-HET females (offspring NIX-HET). In the last case, a couple of females did not get pregnant. We bred rats across two generations: the first generation studied for maternal care consists of heterozygous females of the MAT-HET and μAT-HET epigenotypes, both heterozygous for the DAT gene. MAT-HETs are offspring of a KO male and a WT female, in which the wild allele comes from the maternal lineage (hence the code MAT-HET; see tables by Liberati and colleagues [36]). μAT-HETs are similar in characteristics to MAT-HET females, but they are offspring of a QULL male instead of a KO one (and of a WT female). The QULL group is KO but is also the offspring of a KO female bred with a HET male. As such, the QULL not only has a DAT -/- genotype but also develops while gestating within a hyperdopaminergic uterus [26]. 

The two groups (MAT-HET and μAT-HET females) may show phenotypic differences that originated when the QULL is used (rather than the KO) as sire (i.e., the offspring of μAT-HET dams will also have a KO paternal grandmother). The difference between μAT-HETs and MAT-HETs is that both inherit the truncated allele of DAT from the father, but the former may inherit an allele with additional epigenetic marks (in the μAT-HET group, the QULL rather than KO father is also developed, as a fetus, in the hyperdopaminergic uterus of a KO dam).

The second generation consists of the first generation’s offspring, who were exposed to a series of tests to evaluate the effect of maternal care (related to the genotype) as well as the transgenerational effect. MIX-HET, NIX-HET, and ΜIX-HET are the experimental groups of the second generation. MIX-HETs are the heterozygous offspring of a MAT-HET mother and a KO father; NIX-HETs are the heterozygous offspring of a MAT-HET mother and a QULL father; and μIX-HETs are the offspring of a μAT-HET mother and a KO father. Thus, the putative allele marked by a hyperdopaminergic uterus is inherited in NIX-HETs by coming from the father and in μIX-HETs by coming from a maternal grandfather, of QULL epigenotype.

**Scheme 1:**
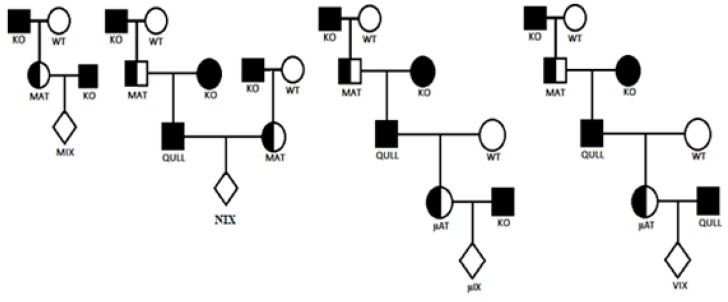
Genealogical tree illustrating the different lines for generation of various heterozygotes epigenotypes. The letter U (QULL) indicates the growth of KO rats in the hyperdopaminergic uterus of their KO dams. The letter M in the code indicates the (grand) maternal origin of the healthy allele (MAT, MIX); the micron letter (micronAT, micronIX) was chosen to maintain the phonetic assonance with the letter M of maternal while being indicative that the (grand) father was a QULL instead of a KO.

In the social interaction test, in marble burying and in Y-maze, we decided to consider two other groups: the WT control group and the vIX combined group. vIXs are similar to MIX-HET but combine the transgenerational origins of both NIX-HET and μIX-HET: vIXs are offspring of a μAT-HET mother and a QULL father and therefore inherit both putatively marked alleles, one from a QULL father and another from a μAT-HET mother (who has a QULL father herself). As a result, a mutated (KO) or also marked (QULL) allele is inherited from the father, while the other mutated (or also marked) allele interacted with the wild allele during the oogenesis of the MAT-HET (or μAT-HET) future mother, respectively [36] (Scheme 1).

### 2.1. Maternal Behavior

#### 2.1.1. Subjects

In the first experiment, we observed 16 lactating mothers, of which 6 were μAT-HET issuing from breeding with a KO male (offspring μIX-HET), 6 were MAT-HET issuing from breeding with a KO male (offspring MIX-HET), and 4 were MAT-HET issuing from breeding with a QULL male (offspring NIX-HET). Mating was planned so that dams gave birth within a window of three days. At birth, pups were culled to 5 ± 1 males and 3 ± 1 females.

#### 2.1.2. Procedures

The analysis of maternal behavior was carried out at postnatal (PND) ages from 3 ± 1 to 14 ± 1, Monday to Thursday for two weeks, with observations divided into three time windows (10:30–11:00, 11:30–12:00, and 12:30–13:00). In each time window, each cage was observed for approximately one minute before proceeding to the remaining cages; three readings were produced every ten minutes for each cage. The behavior of the mother rat towards the pups was verified in the paper ethogram and coded with a thick in the corresponding checkbox (for the ethogram, see Chirico et al., 2017 [38]).

The maternal behaviors explored were: self-grooming, resting, eating, drinking, grooming pups, licking pups, moving pups by mouth, turning with tail in mouth, arcuate breastfeeding, prone breastfeeding, supine breastfeeding, covering pups with body, covering pups with sawdust, digging, and pups breastfeeding themselves while the mother is doing other things. The data collected were entered into a spreadsheet. A sum of the numbers of thicks collected for individual dams was obtained, accounting for each day. These data, divided into epigenotype groups, were entered into the StatView file for final analysis.

### 2.2. Social Interaction during Adolescence

#### 2.2.1. Subjects

The second experiment consists of the observation of adolescent playful interaction in the offspring of experimental dams previously observed for maternal behavior [39]. The subjects are MIX-HETs (offspring of MAT-HET mother and KO father); NIX-HETs (offspring of MAT-HET mother and QULL father); and μIX-HETs (offspring of μAT-HET mother and KO father). MIX-HETs were used as a control group to assess, by comparison, transgenerational effects deriving from either the QULL father (in turn born from the KO grandmother; the crossing of a QULL male with a MAT-HET female generates NIX-HET offspring) or the QULL grandfather (µAT-HET female had a QULL father, and its crossing with a KO male generates µIX-HET offspring).

NIX-HET’s and MIX-HET’s mothers have the same MAT-HET epigenotype; therefore, any difference may result from epigenetic marks at the previous father’s zygote and embryo generation. MIX-HETs and μIX-HETs inherited the healthy allele from their mother and maternal grandmother after it underwent putative modifications through interaction between alleles of the µIX-HET maternal grandmother and grandfather. In MIX-HETs, the allele of grand-sire was just DAT -/- whereas, for µIX-HETs, the truncated allele of grand-sire may have additionally altered due to gestation in the hyperdopaminergic uterus of the maternal great-grandmother.

#### 2.2.2. Procedures

Among the offspring, some were heterozygous and others were KO. Of these, we removed the KO ones during weaning and selected a suitable number of non-sibling heterozygous puppies with the same epigenotype to form the quadruplets. The starting point of the observation was the formation at weaning (PND 22 ± 2) of sixteen yoked quadruplets, each with non-sibling pups of the same epigenotype; for each quadruplet, the male rats are marked with a blue felt tip, with one to three lines on their tail, while the female rat is left unmarked. Thus, rats were marked as “A” (female rat), “B” (single-marked male rat), “C” (double-marked male rat), and “D” (triple-marked male rat). In the initial arrangement, in one cage with the female rat, there was the male rat “B” with a single marking, while in the other yoked cage of the same quadruplet, there were “C” and “D” male rats with double and triple markings.

A 29-day protocol was adopted (start, PND 25; end, PND 55) in which an exchange (“swap”) of the males within each yoked quadruplet took place every three or four days. One male rat in the yoked cage replaced the one in the female rat’s cage, and vice versa, in a rotation schedule where all possible couples were regularly formed to complete the cycle three times.

On the day of the swap, fifteen minutes after the introduction of the swapping male, systematic readings of one minute per cage were taken (the same schedule as for maternal observations, see above). The ethogram used included the following behaviors, divided into:

- Social behaviors: body sniffing, anogenital sniffing, crawling under and over the body of the partner, pushing the partner’s body by crawling between partner and wall, play fighting, allogrooming, and social inactivity.
- Non-social behaviors: cage exploration, rearing, digging, self-grooming, and resting.

At the end of such a period, the female rat per quadruplet was eliminated. The remaining three males per quadruplet were randomly assigned to complete two tests out of a total of three (novelty seeking, social preference, and marble burying).

### 2.3. Novelty Seeking Test

#### 2.3.1. Subjects

The experimental cohort consists of 27 post-adolescent male rats (PND 52 ± 2) divided into MIX-HETs, NIX-HETs, and μIX-HETs (n = 9 per group). No more than two siblings per litter were used. The subjects were tested from 10 a.m. to 4 p.m.

#### 2.3.2. Apparatus and Procedures

The experimental apparatus is an opaque Plexiglas box composed of two different rooms with smooth walls (70 × 30 × 35 cm). The test was performed using a modified version of standard protocols [40]. The door that separates the two rooms can be opened or closed. Both rooms had gray walls and floors, but the wall at the end of one room was white while the one at the end of the other room was black [41]. This test required five consecutive days.

During the habituation phase (from day 1 to day 3), subjects were placed gently in one of the rooms with a closed door for 30 min. During the testing phase (from day 4), the subjects were placed in a familiar room for 5 min and then the door was opened to allow rats to discover and freely explore the other room for 40 min. Videocameras (Sony DCR-SX21E) were placed above the apparatuses to record the whole session. The human experimenters stood in the room but avoided interfering with the ongoing procedure as much as possible. After each session, the floor of the apparatus was cleaned with water and alcohol (1:1).

Observations of video recordings have been performed by “Observer^®^” version 10 (Noldus, NL), a software that calculates the latency, frequency, and duration of several behaviors. Only behaviors related to the unfamiliar room were observed. The monitor screen was divided into four quadrant sections with imaginary lines. We evaluated: exploration, crossing (from one quadrant to another of the same room), rearing, door-sniffing, self-grooming, inactivity, and out of the novel (back to the familiar room).

### 2.4. Social Preference Test

#### 2.4.1. Subjects

The social preference test (SPT) is used to assess social behavior at the age of PND 60 ± 1. The experimental cohort consists of 44 male rats, of which MIX-HET, NIX-HET, and μIX-HET groups have been tested previously with the novelty seeking test; to these, we added 10 that were WT and 10 that were vIX (offspring of μAT-HET mother x QU father). The subjects with vIX and WT genotypes were used as focal rats, and MIX-HET, NIX-HET, and μIX-HET epigenotypes were used as stimulus rats. We evaluated the focal’s choice behavior as related to the stimulus’s epigenotype.

#### 2.4.2. Apparatus and Procedure

The apparatus consisted of a Plexiglass box (70 × 30 × 35 cm) with white and black internal walls and a gray floor. The box consists of two identical chambers separated by a doorway, which allows free access to each chamber. A rectangular metal cage was located at the corner of each chamber, and it was formed by vertical bars (1 cm in width, spaced 1 cm apart). The cage and the whole box were cleaned after every session with water and alcohol (1:1). The test was performed using a modified version of standard protocols [42].

For a period of 24 h before being tested, both stimulus and focal rats underwent a habituation phase. The stimulus rat was placed in the metal cage, without the focal rats around, for 10 min; the focal rats were placed in the box to freely explore it, without the stimulus in the metal cage, for 15 min. Testing occurred repeatedly so that each stimulus epigenotype encountered a similar proportion of WT and vIX focal rats in counterbalanced order. In one of the metal cages, a stimulus rat was gently placed for one minute before inserting the focal one, which was free to explore both chambers and to interact with the metal cage (choosing either the empty one or that with the MIX-HET, μIX-HET, or NIX-HET stimulus rat). The test lasted 30 min.

Three cameras (Sony DCR-SX21E) were placed above the apparatuses to record the session. The human experimenters stood in the room but avoided interfering with the ongoing procedure as much as possible. Video recordings were scored using video processing software “The Observer^®^” version 10 (Noldus, NL). The behavioral analysis includes two types of behavior (frequency and duration): social and non-social. The non-social behaviors are self-grooming, inactivity far from the cage (>2 cm from the cage), exploration, and wall rearing. The social behaviors are allogrooming (attempt), inactivity near the cage (<2 cm from the cage), cage-sniffing, and cage rearing. Each focal rat was tested three times in order to interact with all the possible epigenotypes as a stimulus. The order of stimulus epigenotypes was counterbalanced.

### 2.5. Compulsive Behavior

#### 2.5.1. Marble Burying Test

The apparatus consisted of a Plexiglass box (70 × 30 × 35 cm) with the floor covered with 4 cm of sawdust, in which 15 glass marbles were placed (three lines of five marbles each). Subjects (PND 65 ± 1) were inserted individually into the apparatus and left free to interact with the marbles and the environment for a single 40-min session, after which they were returned to their home cages (with water and food ad libitum). The protocol was taken from [43] and subsequently was adapted by a student (Festucci F., see [44]) in the way we used it. Videocameras (Sony DCR-SX21E) were placed above the apparatuses to record the whole session. The human experimenters stood in the room but avoided interfering with the ongoing procedure as much as possible. Video recordings were scored using video processing software “The Observer^®^” version 10 (Noldus, NL). Sawdust and marbles were cleaned and/or replaced between sessions.

The behavioral analysis includes two types of behavior (frequency and duration): behavior directed toward the environment or objects. Behaviors towards the environment include exploration (of walls and/or sawdust, excluding marbles), digging, self-grooming, and inactivity. Behaviors toward the objects include voluntary and involuntary burying of the marbles, moving the marbles out of their original position, and manipulation of the marbles.

#### 2.5.2. Y-Maze

The apparatus was a labyrinth in the shape of a Y composed of three arms (50 × 10 cm) at a 120° angle from each other and with 20 cm-high walls. The walls were equipped with a removable door slot, allowing one to manually insert panels to open and close each arm separately [45]. Exploiting spontaneously alternating choice behavior in rodents, we focused on perseverant behavior: the more the subject repeatedly chose the same arm of the maze, the more rigid compulsive behavior was developed.

All subjects (PND 70 ± 1) were tested for two consecutive days. At the beginning of each trial, animals were singly placed in a starting arm of the apparatus, whose other arms were closed by the panels. Subsequently, the two panels were simultaneously removed, and subjects were left free to choose which direction to go. Once the choice was made, the panels were resealed, the subjects were returned to the starting arm, and the procedure was repeated. Each subject underwent 21 consecutive trials per day and was subsequently returned to their home cage (with water and food ad libitum).

### 2.6. Statistical Analysis

Behavioral data were analyzed by repeated measures ANOVA (RM-ANOVA) using “StatView^®^” version 5 software (Abacus Concepts, Berkeley, CA, USA). The significance level was set at *p* ≤ 0.05, while the significant trend was set at 0.10 ≤ *p* ≤ 0.05. Post hoc analysis was performed using Tukey’s HSD test. All figures show the standard error of the mean.

For the analysis of maternal behavior, the ANOVA presents a 3 × 2 factorial design in which the “Between” factor represents the epigenotype of the mothers (three levels: MAT-HET (MIX-HET’s mothers) vs. MAT-HET (NIX-HET’s mothers) vs. µAT (µIX-HET’s mothers)) and the “Within” factor is divided into two levels (i.e., the two types of behaviors in contrast).

For the analysis of social interaction, the ANOVA presents a 3 × 2 factorial design, where the “Between” factor represents the epigenotype of the offspring (three levels: MIX-HET vs. NIX-HET vs. µIX-HET) and the “Within” factor is divided into two levels (i.e., the two types of behaviors in contrast).

For the analysis of the circadian cycle, the ANOVA presents a 3 × 24 factorial design, where the “Between” factor represents the epigenotype of the offspring (three levels: MIX-HET vs. NIX-HET vs. µIX-HET) and the “Within” factor represents the 24-hourly points of the average day. In the absence of relevant effects, data are not shown. 

For the analysis of novelty seeking, we used a one-way ANOVA where the “epigenotype” factor represents the epigenotype of offspring (MIX-HET vs. NIX-HET vs. µIX-HET). We analyzed any behavior separately, considering “duration” and\or “frequency”: exploration (only duration), door-sniffing (duration and frequency), crossing (only frequency), time spent in the new chamber (only duration), self-grooming (only duration), inactivity (only duration), and rearing (duration and frequency).

For the analysis of the social preference test, we used a one-way ANOVA where the “epigenotype” factor represents the focal’s epigenotype (WT and vIX). We analyzed any behavior separately, considering “duration” transformed into a percentage, in relation to the chamber with the stimulus (MIX-HET vs. NIX-HET vs. µIX-HET): out of the chamber with stimulus, near cage, cage sniffing, cage rearing, and allogrooming (attempt). For self-grooming and wall-rearing behavior, absolute values were taken.

For the analysis of marble burying, we used a one-way ANOVA where the “epigenotype” factor represents the epigenotype (WT, VIX, MIX-HET, NIX-HET, and µIX-HET). We analyzed any behavior separately, considering “duration”, “frequency” and “latency”: exploration of wall and/or sawdust, excluding marbles (only duration), self-grooming (only duration), inactivity (only duration), voluntary and involuntary burying of the marbles (only frequency and latency), moving of the marbles from their original position (duration, latency, and frequency), manipulation of the marbles (duration, latency, and frequency), and digging (duration, latency, and frequency).

For the analysis of Y-maze, we used a one-way ANOVA where the “epigenotype” factor represents the epigenotype (WT, VIX, MIX-HET, NIX-HET, and µIX-HET). The number of times a subject chose the same direction was transformed into a percentage and compared using repeated-measure ANOVA. The following formula was used to get such a percentage [44,46].

$$\frac{Number\;of\;choices\;in\;the\;same\;direction}{Number\;of\;trials-2}\times 100$$

## 3. Results

### 3.1. Maternal Behavior

**Figure 1:**
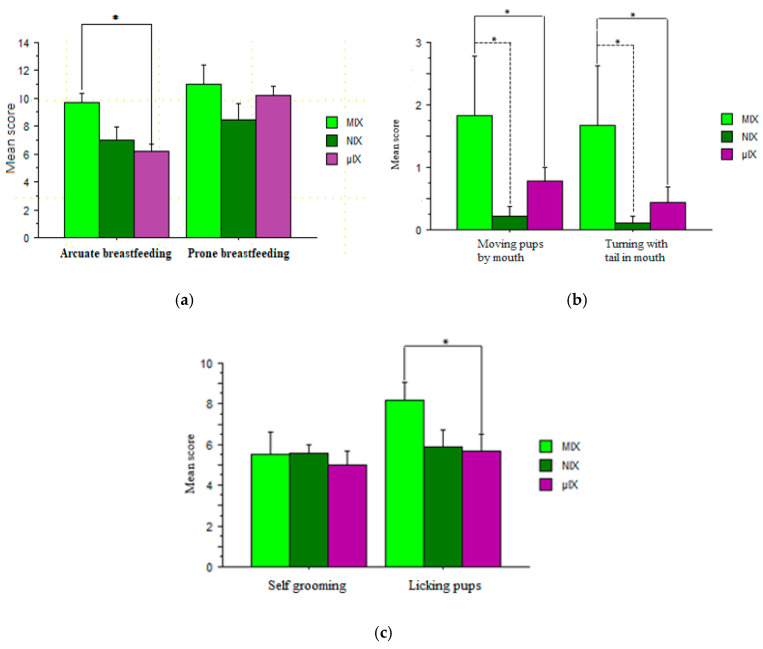
Panel (**a**): Type of lactation: comparison between arcuate and prone breastfeeding in MIX’s dams (MAT), NIX’s dams (MAT), and µIX’s dams (µAT). Panel (**b**): Attention towards the offspring: comparison between moving pups by mouth vs. turning with tail in mouth in MIX’s dams (MAT), NIX’s dams (MAT), and µIX’s dams (µAT). Panel (**c**): Maternal behavior: comparison between self-grooming vs. licking pups in MIX’s dams (MAT), NIX’s dams (MAT), and µIX’s dams (µAT). * significant at *p* ≤ 0.05.

We compared the differential effect of the maternal epigenotypes (MAT-HET vs. µAT-HET) on various maternal behaviors towards their pups (both MIX-HET and NIX-HET are offspring of a MAT-HET mother, while µIX-HET is offspring of a µAT-HET mother). Regarding the type of lactation, namely, arcuate vs. prone, there is a significant interaction (F_2_**_,_**_21_ = 3.963; *p* = 0.0347) with the epigenetics of the mother: a statistically significant effect (Tukey GF = 21; K = 2; Threshold = 3.217) was found in MIX-HET vs. µIX-HET in arcuate breastfeeding but not in prone breastfeeding. We observed how arcuate breastfeeding occurs more often in MIX-HET’s dams (MAT-HET) than in µIX-HET’s dams (µAT-HET), and the former are tendentially (0.10 ≤ *p* ≤ 0.05) elevated over NIX’s dams (MAT-HET). Since we have found no significant effects, prone breastfeeding occurs similarly in all dams. For the arcuate type, a more active and energy-demanding type of lactation, such effort is devoted only towards the MIX-HET rather than the NIX-HET or µIX-HET (Figure 1a). These data may suggest an impairment of maternal behavior when a truncated DAT allele comes from a dam’s father (offspring’s grandfather) or offspring father who has a QULL epigenotype (a DAT -/- itself developed in a hyperdopaminergic uterus).

MIX-HET’s dams are more active in lactation (higher values in arcuate breastfeeding) than NIX’s dams. This differential behavior is not due to the nature of mothers, who have the same epigenotype, but probably to the offspring that may show abnormal behavior (e.g., soliciting less care from dams). In fact, MIX-HET and NIX-HET epigenotypes are both offspring of a MAT-HET dam, but MIX-HETs have a KO father while NIX-HETs have a QULL father (a DAT -/- itself developed in the hyperdopaminergic uterus).

We formally compared the differential effect of the two epigenotypes (MAT-HET vs. µAT-HET) on the type of indirect care towards the pups: moving pups by mouth vs. turning with tail in mouth. Both types of indirect care show a significant interaction (F_2_**_,_**_21_ = 6.561; *p* = 0.0061) with the epigenetics of the mother; as a matter of fact, for both behaviors, we observed how MIX-HET’s dams have a significantly higher profile than NIX-HET’s and µIX-HET’s dams (Tukey GF = 21; K = 2; Threshold = 0.253). For both behaviors, the data confirm that MIX-HET’s dams pay more indirect attention to the offspring than all other dams, while NIX-HET’s dams show the lowest possible values. So, although the epigenotype of the dams is exactly the same (MAT-HET), our results confirm that factors other than the nature of the dams may be causing their lack of interest. Probably, NIX-HET offspring may show abnormal behavior that fails to solicit maternal attention (Figure 1b).

Finally, we formally compared two opposite maternal behaviors: self-grooming vs. licking pups. No significant effect emerged regarding self-grooming. There is no (F_2_**_,_**_21_ = 1.528; *p* = 0.2402) significant interaction, but we tendentially observed (0.10 ≤ *p* ≤ 0.05) more activity in MIX-HET’s dams than in µIX-HET’s and NIX-HET’s dams for licking pups (Figure 1c). Once again, the QULL father or maternal grandfather impairs the licking activity towards the pups (Tukey GF = 21; K = 2; Threshold = 2.42).

### 3.2. Social Interaction during Adolescence

**Figure 2:**
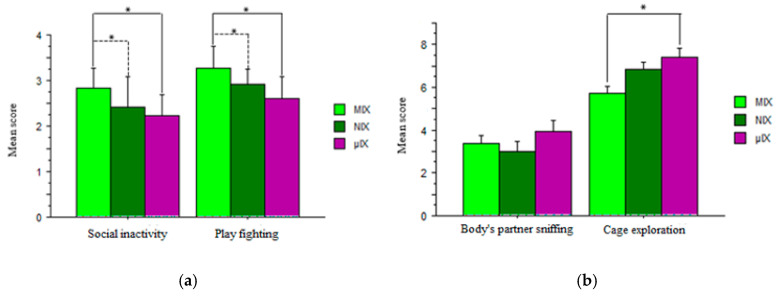
Panel (**a**): Social interaction: comparison between social inactivity and play fighting in MIX vs. NIX vs. µIX. Panel (**b**): Exploration: comparison between social (partner’s body sniffing) and environmental (cage wall or floor exploration) investigations in MIX vs. NIX vs. µIX. * significant at *p* ≤ 0.05.

We compared the differential effects of the three epigenotypes (MIX-HET vs. NIX-HET vs. µIX-HET) on various adolescent behaviors. As for two opposite types of interaction (i.e., social inactivity vs. play fighting), there is a significant interaction (F_2, 45_ = 1.556; *p* = 0.222) with MIX-HETs showing higher values than the other groups (Tukey GF = 45; K = 2; Threshold = 0.108) for both behaviors. So, the reference epigenotype seems to confirm a profile of greater sociability (Figure 2a), both in the passive (staying still but close) and active (rough-and-tumble) forms. NIX-HET and µIX-HET, in contrast, are characterized by less sociability and lower profiles in either of these behaviors. This is in line with the lack of maternal care by their MAT-HET and µAT dams previously described.

We compared the epigenotypes for exploration with a focus on which stimulus attracts their interest, namely, if the subjects prefer exploring a social stimulus through a partner’s body sniffing or exploration of an environmental stimulus like cage walls or sawdust. Our results suggest a significant interaction among the epigenotypes (F_2, 45_ = 2.702; *p* = 0.0780). About cage exploration, µIX-HET showed higher values than MIX-HET (Tukey GF = 45; K = 2; Threshold = 1.564) (Figure 2b), while there are no effects regarding the investigation of the partner. So, data confirm that the phenotype of µIX-HET rats is characterized by increased interest in environmental exploration and much less interest in interacting with the cage mate (both in agonistic and affiliative forms).

### 3.3. Novelty Seeking Test

**Figure 3:**
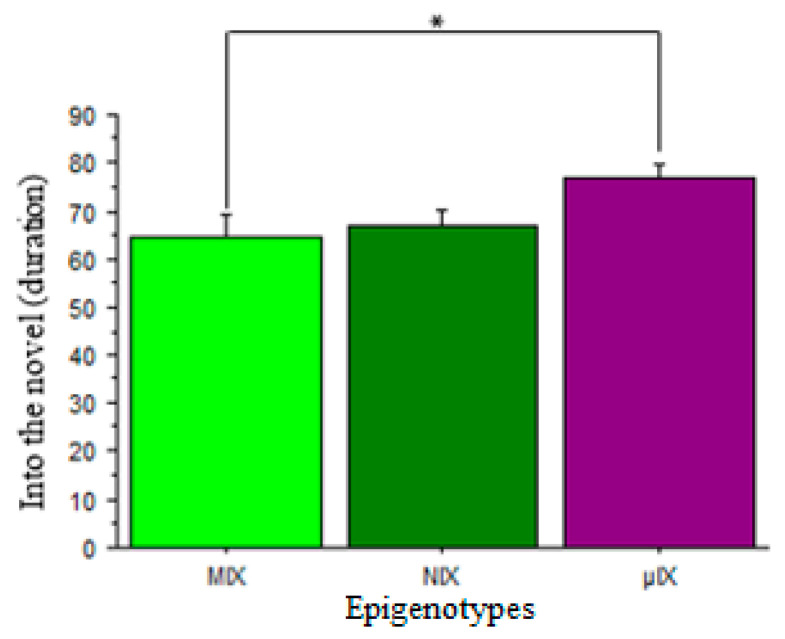
Explorative curiosity: comparison between total duration (%) spent in the new chamber (by MIX vs. NIX vs. µIX). * significant at *p* ≤ 0.05.

We found a significant interaction for the time spent in the unfamiliar chamber among the three epigenetic groups (F_2, 22_ = 2.696; *p* = 0.0897). This unexpected boost in curiosity can be seen in the profiles (Figure 3), where the µIX-HET epigenotype shows significantly higher values than MIX-HET and NIX-HET. This finding confirms attraction by environment in µIX-HET at the expense of social motivation.

### 3.4. Social Preference Test

**Figure 4:**
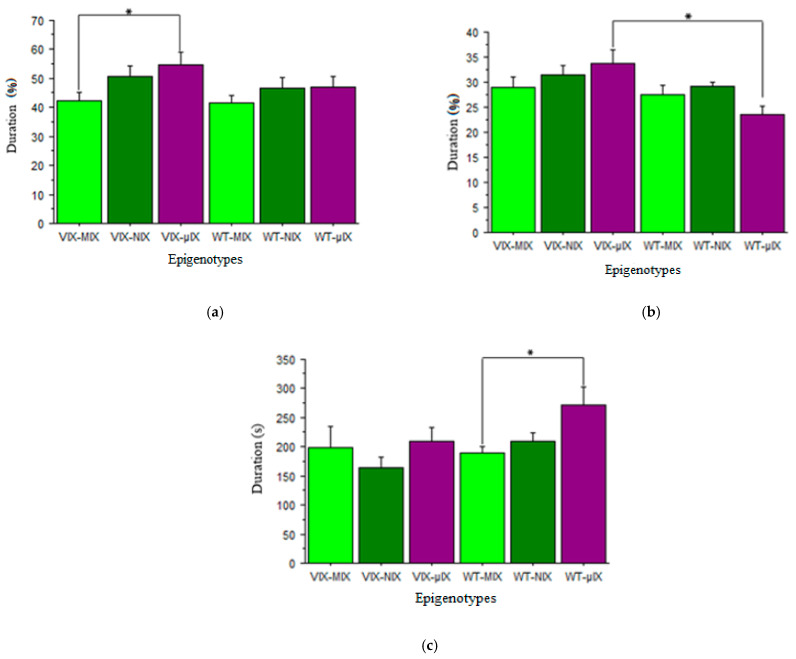
Panel (**a**): Social preference: comparison between the percentage of time spent by vIX vs. WT focal rats near the cage with the stimulus (MIX, NIX, or µIX). Panel (**b**): Comparison between cage sniffing by vIX vs. WT focal rats related to the time spent near the cage. Panel (**c**): Comparison between wall rearing (absolute duration) by vIX vs. WT focal rats. * significant at *p* ≤ 0.05.

To evaluate their sociability, we tested the subjects in a social preference test. We assessed the propensity of a focal rat (vIX vs. WT) to spend time nearby and/or to interact with the cage containing the stimulus rat (MIX-HET, NIX-HET, or µIX-HET). There is a significant interaction of epigenotypes (F_5, 54_ = 2.118; *p* = 0.077) when analyzing time spent near the stimulus cage. vIX focal rats show higher values when the stimulus is an µIX-HET than a MIX-HET; instead, WT focal rats do not show significant differences towards any of the stimulus epigenotypes. So, vIX rats seem really attracted by a stimulus with somewhat similar parental background: the mother’s epigenotype is the same for both (µAT-HET), while the father differs (KO father for µIX-HET offspring and additional impact by a QULL father for vIX offspring) (Figure 4a).

However, the percentage of time spent near the cage may not be relevant alone if focal rats do not confirm interest in exploring socially with the stimulus rat. So, we also compared the percentage of cage sniffing behavior expressed by focal rats, and we found a significant interaction of epigenotype (F_5, 54_ = 3.088; *p* = 0.016): WT and vIX focal rats do not differ when the stimulus is MIX-HET, yet the latter is higher than the former when the stimulus is µIX-HET. WT focals rats did not show differential sniffing between cages containing any epigenotype stimulus, while vIX focals rats are more inclined to interact with the cage when there is an µIX-HET stimulus inside (Figure 4b). This behavior agrees with the results obtained for the time spent near the cage, confirming an anomalous interest expressed by vIX rats towards µIX-HET. Probably, the µIX-HET stimulus could have some anomalous behaviors that attract more vIX focal rats. As such, the attraction near the cage, and the subsequent cage sniffing assume a negative connotation about the interaction between vIX and µIX-HET subjects. So, the µIX-HET’s behavioral anomaly attracts vIX and rejects WT.

We indeed found a significant interaction of epigenotype (F_5, 54_ = 2.258; *p* = 0.061) for wall rearing. WT focal rats show a higher rearing of walls far from the cage, a clear sign of social disinterest, when the stimulus is µIX-HET than when it is MIX-HET. This result is specular and agrees with what was reported previously for the other two parameters. In summary, the epigenotype µIX-HET is not attractive for WT focal rats and/or assumes anomalous behaviors that cause an active disinterest in the latter while generating an active interest in vIX focal subjects (Figure 4c). No significant effects were found in the analysis of allogrooming (attempt) or self-grooming.

### 3.5. Y-Maze

**Figure 5:**
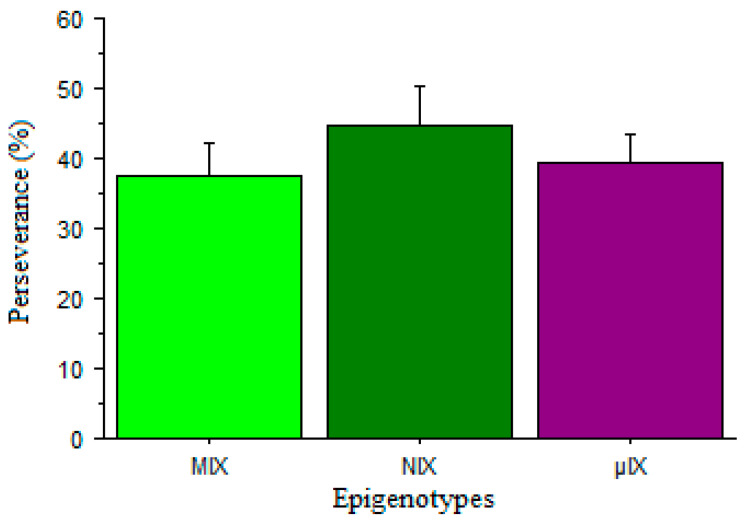
Y-maze: percentage of choice made in the same direction.

The compulsive phenotype was evaluated in terms of rigidity, expressed as a percentage (%) of arm choices made choosing the same direction previously taken. We found a significant tendency (0.10 ≤ *p* ≤ 0.05) among MIX-HET vs. NIX-HET vs. µIX-HET. Our results suggest greater perseverance (% of choices in the same direction) for NIX-HET (almost 45%) than the other two epigenotype groups (levels ≅ 35%) (Figure 5).

### 3.6. Marble Burying Test

We further evaluated the compulsive phenotype among MIX-HET vs. NIX-HET vs. µIX-HET through a marble burying test. Both for rearing (duration) and sniffing (duration), we observed a slight, not significant, tendency (0.10 ≤ *p* ≤ 0.05) with similar profiles for all three epigenotypes. No significant effects were found in the analysis of inactivity (duration). 

**Figure 6:**
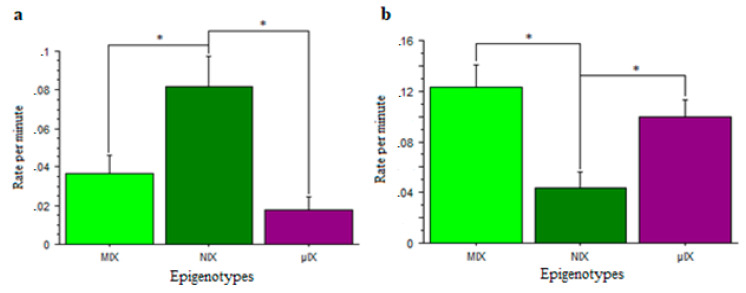

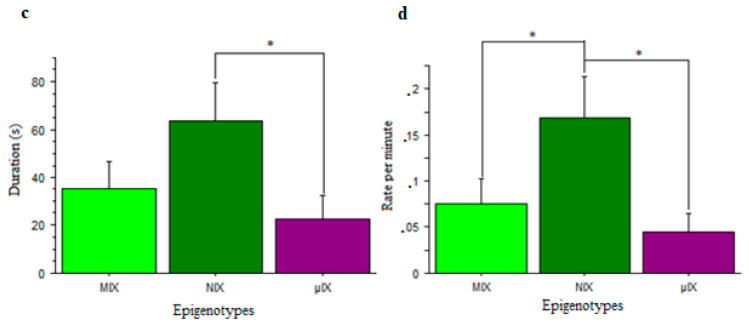
Panels (**a**,**b**): Frequency of voluntary (**a**) and involuntary (**b**) burying of the marbles. Panels (**c**,**d**): Duration (**c**) and frequency (**d**) of the moving of marbles. * significant at *p* ≤ 0.05.

As concerns the frequency of voluntary burying of the marbles, we found a highly significant interaction (F**_2,21_** = 9.431; *p* = 0.0012) among MIX-HET vs. NIX-HET vs. µIX-HET, confirming the net prevalence of this behavior in NIX-HET over the other two epigenotypes (Figure 6a). Consequently, analyzing the frequency of involuntary burying of the marbles, we found a significant interaction (F**_2,21_** = 6.200; *p* = 0.0077) with a specular profile: as expected, NIX-HET showed lower values (about 1/3) than MIX-HET and µIX-HET (Figure 6b). This deliberate burial behavior by NIX-HET would consistently indicate a compulsive phenotype. In contrast, once the marbles are buried, it is less likely to further bury them accidentally.

Considering the moving of marbles, we found a significant interaction both for duration (F_2, 21_ = 2.762; *p* = 0.0861) and for frequency (F_2, 21_ = 4.194; *p* = 0.0293). The most marked effect was between NIX-HET vs. µIX-HET, where values increased in NIX-HET by more than double the time spent doing this behavior. NIX-HET showed higher values, even if not significantly, when compared to MIX-HET (Figure 6c). As concerns frequency, much higher values were observed in NIX-HET rats, more than double compared to the other two epigenotypes (Figure 6d). So, NIX-HET spent in total more time moving the marbles and started to do so more frequently than MIX-HET and µIX-HET, therefore also reducing the average duration of each event.

Observing manipulation of the marbles (i.e., the movement of the marble on the spot), the data revealed a not significant tendency in duration (0.10 ≤ *p* ≤ 0.05) with similar profiles between NIX-HET and µIX-HET and just slightly higher than MIX-HET. The activity of digging (not related to marbles) showed a not significant tendency (0.10 ≤ *p* ≤ 0.05) in duration with little or no significant differences among the groups.

## 4. Discussion

Dopamine is an essential catecholaminergic neurotransmitter implicated in the regulation of the reward system [2], motivation [3], circadian cycle, and working memory [1]. Dopamine, once released, affects many post-synaptic receptors until neurotransmitter removal systems intervene to maintain homeostasis, such as the dopamine transporter (DAT). In order to study the role and function of DAT, different animal models with altered expression of DAT were created.

By means of a stop codon truncating the DAT protein in KO rats, we exploited the functional silencing of the gene encoding for DAT (*SLC6A3*), reducing the expression by about 50% in heterozygous conditions. During growth, the DAT-KO rat is characterized by locomotor disorders, hyperactivity, stereotypes, and impairments in learning and memory [23]. While the phenotypic and behavioral aspects of the DAT-KO model are well known, many questions remain open for the heterozygous condition (DAT-HET). We addressed the complexity of their behavioral repertoire. In fact, the paternal or maternal origin of the healthy allele has a clear impact [27]; additionally, this allele may be sensitive to epigenotypical changes and transgenerational modulations related to the initial ancestor [35,37]. The aim of this work is to evaluate the behavioral effects given by the altered function of DAT in alleles that encountered embryonal development in the hyperdopaminergic uterus and were then inherited by heterozygotes. We found that such additionally hit DAT -/- allele does show sequelae, providing a valid model for the study of pathologies related to the homeostasis of dopamine, such as OCD and ADHD, as well as for the assessment of transgenerational inheritance. In brief, we termed QULL a DAT -/- rat born from a DAT -/- dam. Thus, in addition to our classic breeding [47,48] of WT dam x KO sire and then MAT-HET dam x KO sire, we presently introduced WT dam x QULL sire (whose offspring was termed μAT-HET) and MAT-HET or μAT-HET dam x QULL sire. The experimental subjects consisted of pups issuing from the breeding of a MAT-HET mother with a KO male (offspring MIX-HET), pups issuing from the breeding of a MAT-HET mother with a QULL male (offspring NIX-HET), and pups issuing from the breeding of μAT-HET mother with a KO male (offspring μIX-HET).

### 4.1. Maternal Behavior

MIX-HET’s dams are more active in the arcuate lactation, while μIX-HET’s dams, instead, are more involved in the prone lactation. It’s interesting to observe the profile of NIX-HET’s dams, which not only show reluctance to actively breastfeed but also prefer to explore the environment rather than cure the offspring. This is confirmed by the low values obtained in arcuate lactation and, on the contrary, the high values obtained in involuntary lactation (*pups breastfeeding themselves while the mother is doing other things*). The data collected by evaluating the attention towards the pups (*moving pups by mouth* and *turning with tail in mouth*) confirm the low tendency of NIX-HET’s dams to take care of them, in net contrast with MIX-HET’s dams, which are much more maternal, as can also be seen by the higher values of licking. We assume that the causes behind the lack of care toward the NIX-HET offspring are not to be found in the dams since both MIX-HET and NIX-HET have the same maternal epigenotype (MAT-HET). Nonetheless, the NIX-HET’s dams are more disinterested. We assume that this disinterest may originate in the offspring itself and result from an anomalous behavior shown by the offspring NIX-HET because of their QULL sire. NIX-HET are offspring of a QULL male (a DAT -/- itself developed in a hyperdopaminergic uterus) who aggravates the offspring’s epigenotype through the transmission of further epigenetic modulations. As concerns μAT-HET dams, they show intermediate values between the two other epigenotypes, exhibiting a discrete interest in the offspring’s welfare (*moving pups by mouth* and *turning with tail in mouth)* and an altered propensity for licking. μAT-HET behavior may suggest impairment in maternal care when the truncated DAT allele comes from a dam with a QULL father herself (μAT-HET).

### 4.2. Social Interaction and Social Preference Tests

We evaluated the propensity to play and sociability of the adolescent offspring of previously observed dams. The importance of maternal care, together with epigenetic background, was fully confirmed. The likelihood to interact socially was typical of MIX-HET, and this confirms how maternal care received had a positive effect on the proper development of the offspring. Instead, µIX-HETs show lower interaction with cage mates; this is also reflected in the exploration of walls and sawdust (cage), indicating a high propensity for µIX-HET to explore the environment. This is a coherent profile with less maternal care received from µAT-HET dams by µIX-HET pups. As regards the NIX-HET group, they are not inclined to interact with the cage mate, although the values are higher than µIX-HET. The data collected at adolescence are specular with what is observed in maternal behavior: correct maternal care given to MIX-HET (when pups) by dams is reflected in the high sociality of the grown-up MIX-HET during adolescence; vice versa, NIX-HET’s and µIX-HET’s dams were showing clear-cut weaknesses when taking care of pups, and these are reflected in minimal social interaction towards other adolescent rats. So, our data emphasize the importance of maternal care during the early postnatal period for the development of proper social behavior.

However, the “dam” effect (partially explaining the differences in social behavior) needs its own etiological reason: in our study, we also evaluated the epigenetic factors. We assume that the lower NIX-HET’s and µIX-HET’s sociality may be based on the transgenerational inheritance given by a QULL in the pedigree (NIX-HET’s father and µIX-HET’s maternal grandfather). As a matter of fact, inheriting a DAT-allele also developed in the hyperdopaminergic uterus could aggravate the behavioral phenotype. This concept is consistent with notions put forward just before, namely that the lack of correct maternal care observed in µAT-HET dams could be ascribed to the anomalous allele directly inherited from their own QULL fathers. Conversely, when the same allele was conveyed to the pups, the possible anomalous behavior of NIX-HET offspring was eliciting poor care by MAT-HET dams, which however performed regularly with MIX-HET pups who had no QULL in the pedigree. In conclusion, QULL fathers or maternal grandfathers have clear-cut sequelae.

In the social preference test, we used a WT control group and an vIX group as free-to-move focal subjects, which could choose to interact with the stimulus rat or explore the apparatus. Notably, the vIX epigenotype is a combination of both NIX-HET and µIX-HET, whose phenotype is aggravated by the presence not only of a QULL father but also of a QULL maternal grandfather in the pedigree. This test allowed us to evaluate the potential impact on social behavior of the presence of a QULL epigenotype in the pedigree. 

Both WT and vIX show similar profiles when the social stimulus is a MIX-HET, i.e., standard values of social interaction. When the social stimulus is MIX-HET, focal WTs are very likely to interact with the cage containing the social stimulus and less with the rest of the environment, confirming a normal behavioral phenotype in the WT \ MIX-HET dyad. The data collected in *cage rearing* and *wall rearing* are specular. Despite heterozygous conditions, MIX-HET is a valid reference group for social studies in the DAT-HET [27,49] animal model. When the social stimulus is µIX-HET, conversely, focal WTs have low levels in *cage rearing* and high levels in *wall rearing*, suggesting anomalous behavior by the latter dyad. Instead, when the social stimulus is µIX-HET, the vIX focal group is much more attracted to them. The greater attraction of the vIX focal to the µIX-HET stimulus seems to be confirmed by the higher propensity of the former to interact with the cage containing the social stimulus (*cage sniffing*). We assume that this is an anomalous HET-to-HET attraction due to the presence of a common epigenetic background (both have µAT-HET dams and differ for µIX-HET’s KO father vs. vIX’s QULL father). This would lead the µIX-HET stimulus to express a peculiar phenotype, resulting in avoidance by control WT focal rats yet being remarkably attractive to vIX focal rats. A similar argument is observed when the stimulus is NIX-HET, although with lower values. To summarize the above, there is a likely anomalous behavior shown by µIX-HET stimuli that attracts vIX but rejects WT focal rats.

### 4.3. Y-Maze and Marble Burying Tests

In our work, NIX-HET showed greater perseverance in choosing the same direction during the spontaneous alternation test, indicating a rigid phenotype. This seems to agree with what we found in the marble burying test, which allowed us to evaluate the compulsivity of the epigenotypes we studied. In the comparison between MIX-HET vs. NIX-HET and µIX-HET, we found the highest values for MIX-HET in both exploratory behavior (*rearing* and *sniffing)* and *self-grooming*. MIX-HET and µIX-HET show normal behavior with low voluntary burial and moving of marbles. Such data suggest that these rats show a not-anxious phenotype so that they feel attracted by the surrounding environment without being disturbed by the presence of the marbles. Instead, a compulsive neophobic phenotype emerges from NIX-HET, evident in the specular profiles of *voluntary marble burying* and of *involuntary marble burying*. In fact, the NIX-HET group shows a very high frequency in voluntary burial and a 1/3 lower frequency in involuntary burial. Several studies correlate the burial of marbles with compulsive/obsessive behaviors, which is also confirmed by the decrease in such behaviors after administration of anxiolytic drugs [50,51]. In support of this, the higher values shown by NIX-HET in *moving the marble* and in *manipulating the marble* confirm much more interaction with the marble. In conclusion, NIX-HET, showing hyperactivity and compulsive/obsessive behavior, could represent a valid model to study ADHD and/or OCD. Many studies underline that the proposed neurological pathways downstream of ADHD and OCD are different, but both are characterized by frontostriatal dysfunction; in fact, comorbidity of OCD has been found in patients with ADHD [52,53].

### 4.4. Behavioral Characterization of DAT-HET

While the DAT-KO rat shows gross hyperactivity, overt stereotyped behavior, and social impairments, DAT-HET individuals exhibit a greater variety of behavioral repertoire, which makes them somewhat indifferent to a WT rat. However, HET individuals are not entirely comparable to the WT: the WT individuals exhibit prosocial behavior, indicating appropriate reward processing [44], while the HET individuals display asocial tendencies [49], possibly also modulated by being raised by HET mothers rather than WT mothers [27,54]. HETs are more advantageous from a translational perspective compared to the KO. This advantage comes from the presence of a single functional allele for DAT, rendering them more susceptible to potential epigenetic modulations.

Through various breeding strategies, we were able to assess transgenerational inheritance across generations. Notably, an earlier study [47] demonstrated significant differences between consecutive generations under heterozygous conditions. In MAT-HET individuals, the first encounter within a zygote between the healthy allele and the mutated allele resulted in effects not observed in the mixed offspring issuing from HET x HET breeding. For instance, Carbone’s study [48] highlighted greater locomotor activity in MAT-HET (first generation) compared to the mixed-origin offspring. Consequently, a dam of the WT phenotype appears to confer greater vulnerability to offspring during conception than a dam of the HET phenotype [47,48].

The third generation ([37], termed MYX-HET) appears to be more active socially and locomotor-wise compared to the second generation, corresponding to MIX-HET, μIX-HET, and NIX-HET. This is possibly indicating that the vulnerability resulting from the initial (WT × KO) cross affected the second generation but disappeared or diminished in subsequent generations.

### 4.5. Future Perspectives and Limits

In our study, we assessed the effect of allelic distribution along the pedigree on the behavioral phenotype of three DAT-HET experimental groups. Despite all groups being heterozygous, significant variations in behavior emerged. We hypothesized [36] that differences may be related to the inheritance of the healthy/mutated allele with its epigenetic putative modulations. In combination with maternal care received during the postnatal period, epigenetic marks on either allele contribute to the gene-environment interaction, resulting in varying outcomes among different heterozygotes. Indeed, maternal influence is known to be important in establishing stress response mechanisms in offspring [55,56,57]. Mother–pup contact in rats primarily occurs in the context of a nest, when the mother approaches the litter and engages in lactation with various degrees of effort as well as in licking and grooming behaviors towards her pups [58]. The effect of maternal care was clearly observed in the behavior of NIX-HET (offspring of a MAT-HET mother and a QULL father) and μIX-HET (offspring of a μAT-HET mother and a KO father): in both cases, maternal care was altered or reduced. Yet, the former developed a compulsive/obsessive phenotype, while the latter exhibited a socially depressive phenotype.

This study explored potential transgenerational inheritance along with the impact of maternal care to better understand the epigenetic changes resulting from gene-environment interactions. It emerged that the influence on maternal care could be directly prompted by QULL ancestors in her pedigree (μAT-HET dams) or even indirectly, by altering attraction to pups if their mutated allele is coming from the QULL sire (see MAT-HET dams with NIX-HET pups). This played a significant role in the behavioral development of the offspring, leading to different phenotypes. However, the assessment of maternal and pups’ behavior represented a limitation of our study since our observations were limited to a non-continuous time period (a few one-minute samples over two weeks). To further investigate the impact of maternal behavior correlated with epigenotypes, we could potentially adopt a continuous recording of ultrasounds emitted by pups and of mother–offspring interactions in future studies [59].

Furthermore, we acknowledge the absence of molecular investigations on potential epigenetic targets; future studies need to explore adaptations at the promoter of DAT in alleles of both lines in order to better distinguish the contribution of maternal care and epigenetic inheritance to the behavioral phenotype of the offspring.

## 5. Conclusions

The underlying factors of neuropsychiatric disorders are not clear yet, but it is known [60] that genetic factors play an important role in the onset of conditions like ADHD, ASD, and OCD. Besides the genetic component, the environment can potentially increase vulnerability to these conditions by interacting with genes. Among environmental factors, we can include the cell context in which gametes mature, namely the epididymis and uterus, plus a parent-of-origin effect (POE): effects passed from parents to offspring lead to a phenotype that can depend on either paternal or maternal transmission [61]. Several studies have demonstrated the asymmetrical parental transmission of genes associated with these pathologies, such as *SLC6A3*, DRD4 and DRD5 [62,63], which exhibit paternal over-transmission. Our present model enables epigenetic-transmission studies and track an initial POE across generations and different types of backcrosses. Despite the fact that DAT +/- rats resulting from these crosses should be seen as genetically identical individuals, because of their pedigree they may exhibit epigenetic variations: these are largely neglected but entail increased susceptibility to neuropsychiatric disorders.

Notably, the crucial difference in either case (NIX-HET or μIX-HET) was the QULL ancestor in the pedigree, which acted once as a direct sire and once as maternal grand-sire, respectively. When evaluating the effect of the QULL epigenotype (DAT -/- epigenotype, raised in a hyperdopaminergic uterine environment), which is common to both the NIX-HET and μIX-HET groups, this concept emerged: the “mother” effect is not limited to maternal care alone but also extends to the gestation period. In fact, the uterine environment not only influences the direct offspring during pregnancy but also impacts the developing germ cells of the gestating offspring, thereby affecting future generations [28]. In NIX-HET rats, the DAT-KO grand-dam exerted such sequelae via its male offspring (QULL rats then becoming sires); in μIX-HET rats, the DAT-KO great-grand dam did so via her QULL males, whose sperm conceived a μAT-HET female embryo. Although both are born from WT dams, the μAT-HET clearly differ from MAT-HET rats when becoming dams at their turn.

Although it is challenging to distinguish between changes resulting from epigenetic inheritance and changes resulting from evident differences in maternal care, previous studies [27] suggest that the parental origin of the DAT allele may carry more sequelae than maternal care itself. Indeed, our study has highlighted that putative epigenetic changes originating within a compromised uterine environment and thereafter transmitted through the egg or sperm (in combination with stressful environmental factors such as altered maternal care) can alter the epigenetic phenotype of the offspring [64].

Our conclusion is that, while the MIX-HET group, lacking a QULL ancestor and receiving proper maternal care, displayed normal behavior, the inheritance transmitted by the QULL epigenotype, in combination with different maternal care, manifested in two different behavioral phenotypes in NIX-HET and μIX-HET: we found persistent behavioral effects that varied from compulsive and rigid to depressive and anxious phenotypes, in line with previous studies [65,66].

## Data Availability

The original data pertaining to this paper are stored on a computer located at ISS (building 19, room D-11) in the office of the corresponding author. All raw data can be made available upon request.

## References

[1] Luciana M., Collins P.F., Depue R.A. (1998). Opposing roles for dopamine and serotonin in the modulation of human spatial working memory functions. Cereb. Cortex.

[2] Schultz W. (1998). Predictive reward signal of dopamine neurons. J. Neurophysiol..

[3] Salamone J.D., Correa M. (2012). The mysterious motivational functions of mesolimbic dopamine. Neuron.

[4] Chandler D.J., Waterhouse B.D., Gao W.-J. (2014). New perspectives on catecholaminergic regulation of executive circuits: Evidence for independent modulation of prefrontal functions by midbrain dopaminergic and noradrenergic neurons. Front. Neural Circuits.

[5] Stolzenberg D.S., Champagne F.A. (2016). Hormonal and non-hormonal bases of maternal behavior: The role of experience and epigenetic mechanisms. Horm. Behav..

[6] Numan M., Stolzenberg D.S. (2009). Medial preoptic area interactions with dopamine neural systems in the control of the onset and maintenance of maternal behavior in rats. Front. Neuroendocr..

[7] Shahrokh D.K., Zhang T.-Y., Diorio J., Gratton A., Meaney M.J. (2010). Oxytocin-dopamine interactions mediate variations in maternal behavior in the rat. Endocrinology.

[8] Hansen S., Bergvall A.H., Nyiredi S. (1993). Interaction with pups enhances dopamine release in the ventral striatum of maternal rats: A microdialysis study. Pharmacol. Biochem. Behav..

[9] Champagne F.A., Chretien P., Stevenson C.W., Zhang T.Y., Gratton A., Meaney M.J. (2004). Variations in nucleus accumbens dopamine associated with individual differences in maternal behavior in the rat. J. Neurosci..

[10] Afonso V.M., Grella S.L., Chatterjee D., Fleming A.S. (2008). Previous maternal experience affects accumbal dopaminergic responses to pup-stimuli. Brain Res..

[11] Afonso V.M., King S., Chatterjee D., Fleming A.S. (2009). Hormones that increase maternal responsiveness affect accumbal dopaminergic responses to pup-and food-stimuli in the female rat. Horm. Behav..

[12] Afonso V.M., King S.J., Novakov M., Burton C.L., Fleming A.S. (2011). Accumbal dopamine function in postpartum rats that were raised without their mothers. Horm. Behav..

[13] Afonso V.M., Shams W.M., Jin D., Fleming A.S. (2013). Distal pup cues evoke dopamine responses in hormonally primed rats in the absence of pup experience or ongoing maternal behavior. J. Neurosci..

[14] Atzil S., Gao W., Fradkin I., Barrett L.F. (2018). Growing a social brain. Nat. Hum. Behav..

[15] Rice M.E., Cragg S.J. (2008). Dopamine spillover after quantal release: Rethinking dopamine transmission in the nigrostriatal pathway. Brain Res. Rev..

[16] Cragg S.J., Rice M.E. (2004). DAncing past the DAT at a DA synapse. Trends Neurosci..

[17] Agid O., Lerer B. (1999). Risperidone augmentation of paroxetine in a case of severe, treatment-refractory obsessive-compulsive disorder without comorbid psychopathology. J. Clin. Psychiatry.

[18] Ashok A.H., Mizuno Y., Volkow N.D., Howes O.D. (2017). Association of stimulant use with dopaminergic alterations in users of cocaine, amphetamine, or methamphetamine: A systematic review and meta-analysis. JAMA Psychiatry.

[19] DiCarlo G.E., Aguilar J.I., Matthies H.J., Harrison F.E., Bundschuh K.E., West A., Hashemi P., Herborg F., Rickhag M., Chen H. (2019). Autism-linked dopamine transporter mutation alters striatal dopamine neurotransmission and dopamine-dependent behaviors. J. Clin. Investig..

[20] Mazei-Robison M.S., Couch R.S., Shelton R.C., Stein M.A., Blakely R.D. (2005). Sequence variation in the human dopamine transporter gene in children with attention deficit hyperactivity disorder. Neuropharmacology.

[21] Mazei-Robison M.S., Bowton E., Holy M., Schmudermaier M., Freissmuth M., Sitte H.H., Galli A., Blakely R.D. (2008). Anomalous dopamine release associated with a human dopamine transporter coding variant. J. Neurosci..

[22] Robertson S.D., Matthies H.J.G., Galli A. (2009). A closer look at amphetamine-induced reverse transport and trafficking of the dopamine and norepinephrine transporters. Mol. Neurobiol..

[23] Leo D., Sukhanov I., Gainetdinov R.R. (2018). Novel translational rat models of dopamine transporter deficiency. Neural Regen. Res..

[24] Cyr M., Beaulieu J.-M., Laakso A., Sotnikova T.D., Yao W.-D., Bohn L.M., Gainetdinov R.R., Caron M.G. (2003). Sustained elevation of extracellular dopamine causes motor dysfunction and selective degeneration of striatal GABAergic neurons. Proc. Natl. Acad. Sci. USA.

[25] Tremolizzo L., Carboni G., Ruzicka W.B., Mitchell C.P., Sugaya I., Tueting P., Sharma R., Grayson D.R., Costa E., Guidotti A. (2002). An epigenetic mouse model for molecular and behavioral neuropathologies related to schizophrenia vulnerability. Proc. Natl. Acad. Sci. USA.

[26] Manoni G., Puzzo C., Gigantesco A., Adriani W. (2022). Behavioral Phenotype in Heterozygous DAT Rats: Transgenerational Transmission of Maternal Impact and the Role of Genetic Asset. Brain Sci..

[27] Oggiano M., Buccheri C., Alleva E., Adriani W. (2022). Dopaminergic modulation of the circadian activity and sociability: Dissecting parental inheritance versus maternal styles as determinants of epigenetic influence. Behav. Brain Res..

[28] Lacal I., Ventura R. (2018). Epigenetic Inheritance: Concepts, Mechanisms and Perspectives. Front. Mol. Neurosci..

[29] Kaufman J., Zigler E., Gelles R.J., Loseke D.R. (1993). The intergenerational transmission of abuse is overstated. Current Controversies on Family Violence.

[30] Mathews C.A., Kaur N., Stein M.B. (2008). Childhood trauma and obsessive-compulsive symptoms. Depress. Anxiety.

[31] Cohen P., Brown J., Smailes E. (2001). Child abuse and neglect and the development of mental disorders in the general population. Dev. Psychopathol..

[32] Kroska E., Miller M., Roche A.I., Kroska S.K., O’Hara M.W. (2018). Effects of traumatic experiences on obsessive-compulsive and internalizing symptoms: The role of avoidance and mindfulness. J. Affect. Disord..

[33] Pears K.C., Capaldi D.M. (2001). Intergenerational transmission of abuse: A two-generational prospective study of an at-risk sample. Child Abus. Negl..

[34] Oliver J.E. (1993). Intergenerational transmission of child abuse: Rates, research, and clinical implications. Am. J. Psychiatry.

[35] Puzzo C., D’Angiò R., Albanese S., Orlando D., Mangili I., Capobianco M., Liberati A.S., Adriani W. (2023). Inheritance of wild and truncated DAT alleles from grand-parents: Opposite transgenerational consequences on the behavioral phenotype in adolescent DAT heterozygous rats. Neurosci. Lett..

[36] Liberati A.S., Calcaprina B., Adriani W. (2022). Keeping Track of the Genealogy of Heterozygotes Using Epigenetic Reference Codes and Breeding Tables. Front. Behav. Neurosci..

[37] Puzzo C., Festucci F., Curcio G., Gigantesco A., Adriani W. (2023). Exploring Transgenerational Inheritance in Epigenotypes of DAT Heterozygous Rats: Circadian Anomalies and Attentional Vulnerability in the Phenotype. Master Thesis of Professional Stage for Psychologists (under revision). Master’s Thesis.

[38] Chirico D., Romano E., Famele M., Draisci R., Mancinelli R., Pascucci T., Adriani W. (2017). Forced but not free-choice nicotine during lactation alters maternal behaviour and noradrenergic system of pups: Impact on social behaviour of adolescent isolated male rats. Neuroscience.

[39] File S.E., Hyde J. (1978). Can social interaction be used to measure anxiety?. Br. J. Pharmacol..

[40] Bardo M., Bevins R. (2000). Conditioned place preference: What does it add to our preclinical understanding of drug reward?. Psychopharmacol..

[41] Beaudet G., Paizanis E., Zoratto F., Lacivita E., Leopoldo M., Freret T., Laviola G., Boulouard M., Adriani W. (2017). LP-211, a selective 5-HT7 receptor agonist, increases novelty-preference and promotes risk-prone behavior in rats. Synapse.

[42] Moy S.S., Nadler J.J., Perez A., Barbaro R.P., Johns J.M., Magnuson T.R., Piven J., Crawley J.N. (2004). Sociability and preference for social novelty in five inbred strains: An approach to assess autistic-like behavior in mice. Genes Brain Behav..

[43] Jimenez-Gomez C., Osentoski A., Woods J.H. (2011). Pharmacological evaluation of the adequacy of marble burying as an animal model of compulsion and/or anxiety. Behav. Pharmacol..

[44] Festucci F., Annunzi E., Pepe M., Curcio G., D’Addario C., Adriani W. (2022). Dopamine-transporter heterozygous rats carrying maternal wild-type allele are more vulnerable to the development of compulsive behavior. Synapse.

[45] Conrad C.D., Galea L.A.M., Kuroda Y., McEwen B.S. (1996). Chronic stress impairs rat spatial memory on the Y maze, and this effect is blocked by tianeptine pretreatment. Behav. Neurosci..

[46] Rashid M.H., Sparrow N.A., Anwar F., Guidry G., Covarrubias A.E., Pang H., Bogguri C., Karumanchi S.A., Lahiri S. (2021). Interleukin-6 mediates delirium-like phenotypes in a murine model of urinary tract infection. J. Neuroinflammation.

[47] Pepe M., Calcaprina B., Vaquer F., Laviola G., Adriani W. (2022). Truncated dopamine transporter’s epigenetics: Heterozigosity of the grandmother rat temperates the vulnerable phenotype in second-generation offspring. Int. J. Dev. Neurosci..

[48] Carbone C., Brancato A., Adinolfi A., Russo S.L.M.L., Alleva E., Cannizzaro C., Adriani W. (2020). Motor Transitions’ Peculiarity of Heterozygous DAT Rats When Offspring of an Unconventional KOxWT Mating. Neuroscience.

[49] Adinolfi A., Zelli S., Leo D., Carbone C., Mus L., Illiano P., Alleva E., Gainetdinov R.R., Adriani W. (2019). Behavioral characterization of DAT-KO rats and evidence of asocial-like phenotypes in DAT-HET rats: The potential involvement of norepinephrine system. Behav. Brain Res..

[50] Njung’e K., Handley S.L. (1991). Evaluation of marble-burying behavior as a model of anxiety. Pharmacol. Biochem. Behav..

[51] Gyertyán I. (1995). Analysis of the marble burying response: Marbles serve to measure digging rather than evoke burying. Behav. Pharmacol..

[52] Abramovitch A., Dar R., Hermesh H., Schweiger A. (2012). Comparative neuropsychology of adult obsessive-compulsive disorder and attention deficit/hyperactivity disorder: Implications for a novel executive overload model of OCD. J. Neuropsychol..

[53] Norman L.J., Carlisi C., Lukito S., Hart H., Mataix-Cols D., Radua J., Rubia K. (2016). Structural and Functional Brain Abnormalities in Attention-Deficit/Hyperactivity Disorder and Obsessive-Compulsive Disorder: A Comparative Meta-analysis. JAMA Psychiatry.

[54] Mariano S., Pardo M., Buccheri C., Illiano P., Adinolfi A., Russo S.L.M.L., Alleva E., Carbone C., Adriani W. (2020). Own or dam’s genotype? Classical colony breeding may bias spontaneous and stress-challenged activity in DAT-mutant rats. Dev. Psychobiol..

[55] Levine S. (1994). The ontogeny of the hypothalamic-pituitary-adrenal axis. The influence of maternal factors. Ann. N. Y. Acad. Sci..

[56] Fleming A.S., O’Day D.H., Kraemer G.W. (1999). Neurobiology of mother-infant interactions: Experience and central nervous system plasticity across development and generations. Neurosci. Biobehav. Rev..

[57] Meaney M.J. (2001). Maternal care, gene expression, and the transmission of individual differences in stress reactivity across generations. Annu. Rev. Neurosci..

[58] Stern J.M. (1997). Offspring-induced nurturance: Animal-human parallels. Dev. Psychobiol..

[59] Ferreira A., Agrati D., Uriarte N., Pereira M., Zuluaga M.J., Cruz-Morales S.E., Pedro Arriaga-Ramírez J.C. (2012). The rat as a model for studying maternal behavior. Behavioral Animal Models.

[60] Pettersson E., Larsson H., Lichtenstein P. (2015). Common psychiatric disorders share the same genetic origin: A multivariate sibling study of the Swedish population. Mol. Psychiatry.

[61] Rampersaud E., Mitchell B.D., Pollin T.I., Fu M., Shen H., O’connell J.R., Ducharme J.L., Hines S., Sack P., Naglieri R. (2008). Physical activity and the association of common FTO gene variants with body mass index and obesity. Arch. Intern. Med..

[62] Hawi Z., Kent L., Hill M., Anney R., Brookes K., Barry E., Franke B., Banaschewski T., Buitelaar J., Ebstein R. (2010). ADHD and DAT1: Further evidence of paternal over-transmission of risk alleles and haplotype. Am. J. Med. Genet. Part B Neuropsychiatr. Genet..

[63] Hawi Z., Segurado R., Conroy J., Sheehan K., Lowe N., Kirley A., Shields D., Fitzgerald M., Gallagher L., Gill M. (2005). Preferential transmission of paternal alleles at risk genes in attention-deficit/hyperactivity disorder. Am. J. Hum. Genet..

[64] Hackett J.A., Sengupta R., Zylicz J.J., Murakami K., Lee C., Down T.A., Surani M.A. (2013). Germline DNA Demethylation Dynamics and Imprint Erasure Through 5- Hydroxymethylcytosine. Science.

[65] Ly L., Chan D., Trasler J.M. (2015). Developmental windows of susceptibility for epigenetic inheritance through the male germline. Semin. Cell Dev. Biol..

[66] Johnson F.K., Delpech J.-C., Thompson G.J., Wei L., Hao J., Herman P., Hyder F., Kaffman A. (2018). Amygdala hyper-connectivity in a mouse model of unpredictable early life stress. Transl. Psychiatry.

